# Common and distinct functions of mouse Dot1l in the regulation of endothelial transcriptome

**DOI:** 10.3389/fcell.2023.1176115

**Published:** 2023-06-15

**Authors:** Hyunjin Yoo, Hyeonwoo La, Chanhyeok Park, Seonho Yoo, Hyeonji Lee, Hyuk Song, Jeong Tae Do, Youngsok Choi, Kwonho Hong

**Affiliations:** Department of Stem Cell and Regenerative Biotechnology, Institute of Advanced Regenerative Science, Konkuk University, Seoul, South Korea

**Keywords:** blood endothelial cell, lymphatic endothelial cell, epigenetics, DOT1l, transcriptional regulation

## Abstract

Epigenetic mechanisms are mandatory for endothelial called lymphangioblasts during cardiovascular development. Dot1l-mediated gene transcription in mice is essential for the development and function of lymphatic ECs (LECs). The role of Dot1l in the development and function of blood ECs blood endothelial cells is unclear. RNA-seq datasets from Dot1l-depleted or -overexpressing BECs and LECs were used to comprehensively analyze regulatory networks of gene transcription and pathways. Dot1l depletion in BECs changed the expression of genes involved in cell-to-cell adhesion and immunity-related biological processes. Dot1l overexpression modified the expression of genes involved in different types of cell-to-cell adhesion and angiogenesis-related biological processes. Genes involved in specific tissue development-related biological pathways were altered in Dot1l-depleted BECs and LECs. Dot1l overexpression altered ion transportation-related genes in BECs and immune response regulation-related genes in LECs. Importantly, Dot1l overexpression in BECs led to the expression of genes related to the angiogenesis and increased expression of MAPK signaling pathways related was found in both Dot1l-overexpressing BECs and LECs. Therefore, our integrated analyses of transcriptomics in Dot1l-depleted and Dot1l-overexpressed ECs demonstrate the unique transcriptomic program of ECs and the differential functions of Dot1l in the regulation of gene transcription in BECs and LECs.

## 1 Introduction

The blood endothelial cells (BECs) line the inner cavity of the vascular system and provide a physical barrier, transportation of molecules and oxygen, and immune responses (Galley and Webster, 2004; McCarron et al., 2017). Although, in general, BECs originate from mesodermal lineages, ample evidence suggests that multiple developmental stages- and/or organ-type-specific sub-mesoderm origins of BECs exist. The molecular, functional, and structural heterogeneity in different types of ECs has been identified (Aird, 2007a; Aird, 2007b; Aird, 2012; Jambusaria et al., 2020; Becker et al., 2022; Hou et al., 2022).

Early studies proposed that a specialized mesoderm-origin cell, called a hemangioblast, differentiates into hematopoietic stem cells and angioblasts after gastrulation. Subsequently, angioblasts generate BEC progenitors (Chung et al., 2002). In mice, embryonic vasculatures arise simultaneously in embryonic and extraembryonic tissues via vasculogenesis, a process of *de novo* blood vessel formation (Drake and Fleming, 2000; Chong et al., 2011). After that, BECs respond to angiogenic growth factors, sprouting from pre-existing vessels by angiogenesis (Majesky, 2018) and undergo specialization into venous or arterial BECs before the formation of mature vasculature by diverse factors, such as hemodynamic forces and transcriptional programs (Jain, 2003; Heil et al., 2006; Lin, 2007) After arterial or venous BEC fate is determined, each BEC expresses distinct sets of transcriptomes and displays different branching capacities (Jain, 2003; Torres-Vazquez et al., 2003). Following the maturation of blood vessels, some of the venous ECs in the anterior part of the cardinal vein (CV) express Prox1, Lyve1, and Sox18, and differentiation to lymphatic EC (LECs) begins at approximately embryonic day (E) 9–10 in mice (Tammela and Alitalo, 2010; Potente and Makinen, 2017). Accumulating evidence suggests that LECs of developing mouse organs have heterogeneity in both cellular origin and function (Klotz et al., 2015; Martinez-Corral et al., 2015; Pichol-Thievend et al., 2018; Maruyama et al., 2019; Lioux et al., 2020; Aquino et al., 2021). Furthermore, Prox1 (+) LECs in the CV are differentiated from Pax3 (+) paraxial mesoderm-derived LEC progenitor cells (called lymphangioblasts) in mouse (Stone and Stainier, 2019; Lupu et al., 2022).

During the development of BECs and LECs, various epigenetic mechanisms, including DNA methylation and histone modifications are involved in EC development and functions (Gutierrez et al., 2021; Yu et al., 2021). Furthermore, there are epigenetic differences in ECs depending on organotypic and developmental origin factors (Bronneke et al., 2012; Nakato et al., 2019; Gutierrez et al., 2021; Tacconi et al., 2021). However, epigenetic modifiers establishing vessel type-specific epigenetic landscapes that contribute to the development of unique transcriptomic signatures during development remain elusive.

Dot1l is the only known histone H3K79 methyltransferase that produces mono-, di-, tri-methyl H3K79 (H3K79me1-3) (Feng et al., 2002). Dot1l has diverse roles in biological processes, including DNA repair (Huyen et al., 2004), transcription elongation (Mueller et al., 2007), telomeric silencing (Jones et al., 2008), regulation of the cell cycle (Jones et al., 2008), pluripotent stem cell differentiation (Barry et al., 2009), and immune responses (Kealy et al., 2020). Dot1l is essential for embryonic blood vessel, heart, and lymphatic vessel development and functions via transcriptional regulation of lymphatic genes (Jones et al., 2008; Nguyen et al., 2011; Yoo et al., 2020). Dot1l loss in BECs leads to decreased tube formation and sprouting *in vitro* and vessel network formation *in vivo* (Duan et al., 2016). Our group previously showed that Dot1l loss in both BECs and LECs causes severe defects in LEC development and function. In particular, we revealed functional heterogeneity of Dot1l in LEC defects depending on the developmental origin and organotypes of LECs (Yoo et al., 2020). However, the regulatory mechanism by which Dot1l functions differentially in BECs vs. LECs at the molecular level remains unclear.

Using genome-scale transcriptomic analysis, this study showed that mouse Dot1l regulates common or distinct transcriptomic programs in an EC type-dependent manner, providing background information for identifying factors orchestrating the transcriptional change together with Dot1l and conditional target specificity of Dot1l. Furthermore, since BECs and LECs branch off from the same source (Wigle and Oliver, 1999; Srinivasan et al., 2007; Francois et al., 2012) and have distinguishable phenotypes, this study may provide insights into the cell fate decision mechanism and the function gaining process of these cell types.

This study found that Dot1l loss changes the expression of genes involved in lipid metabolism in BECs and the chemotaxis in LECs. Meanwhile, Dot1l overexpression alters the expression of genes involved in ion transportation and immune response regulation in both BECs and LECs. Taken together, these findings reveal a distinct Dot1l function in developing BECs and LECs and the heterogeneity of the transcriptomic program in the ECs.

## 2 Materials and methods

### 2.1 Animals and isolation of skin BECs

The endothelial-specific Dot1l conditional knockout mouse has been described previously (Yoo et al., 2020). Briefly, E15.5 Tie2-Cre(+); Dot1l^2f/2f^ (cKO), and Tie2-Cre(−); Dot1l^2f/2f^ (Cont) embryo skins were harvested and dissociated in media containing type II and IV collagenase and DNase I (LS006333; Worthington Biochemical Corp.) for 20 min at 37°C. Cells that passed through 40-µm cell strainers were incubated with anti-CD45 and anti-F4/80 antibodies (13–0451 and 13–4801, eBioscience) for 1 h at room temperature to deplete macrophages. F4/80 (−)/CD45 (−) cells were collected and incubated with Lyve1 antibody (13–0443, eBioscience) to collect LECs. Together with the LECs, isolated F4/80 (−)/CD45 (−)/Lyve1 (−) BECs were subjected to RT-qPCR or RNA-seq analysis. All animal studies were reviewed and approved by the Institute of Animal Care and Use Committee (IACUC) of Konkuk University (IACUC#KU18027).

### 2.2 Lentivirus-mediated Dot1l overexpression in BECs and LECs

The overexpression of Dot1l has been described in a previous study (Yoo et al., 2020). Briefly, guide RNAs targeting the Dot1l promoter were designed and subcloned into the BbsI sites of the vectors (cat. #53186, #53187, #53188, and #53189, Addgene). The vectors were then cloned into a lentivirus vector (59791, Addgene) containing catalytically dead Cas9 (dCas9) fused with VP64, guide RNAs targeting Dot1l promoter regions and enhanced green fluorescent protein genes (Figure 3A). The Dot1l overexpression vector with lentiviral packaging vectors [psPAX2 (cat. #12260, Addgene) and pMD2.G (cat. #12259, Addgene) vectors] was transfected into HEK293T cells to produce lentiviral particles. Mouse embryonic dermal LECs (C57-6064L, Cell Biologics) and embryonic yolk sac BECs (CRL-2581, ATCC) were used for viral transduction. Each cell line was maintained according to the manufacturer’s instructions. Once ECs reached approximately 50% confluency, viral particles were added to BECs and LECs for Dot1l overexpression. ECs transduced with empty lentivirus were used as controls. EGFP(+) cells were sorted using FACS Aria (BD Biosciences) and used for RT-qPCR and RNA-sequencing analysis.

### 2.3 RNA isolation and quantitative RT-PCR (RT-qPCR)

Dot1l cKO ECs from E15.5 skins and cultured Dot1l overexpression ECs were used for RT-qPCR. Total RNAs were extracted using RNeasy Plus Mini Kit (Qiagen). SMARTer Pico PCR cDNA synthesis kit (Takara) along with Advantage 2 PCR Kit (Takara) and TOPscript RT DryMix cDNA synthesis kit (Enzynomics) were used for cDNA synthesis of Dot1l cKO ECs and Dot1l overexpression ECs, respectively. RT-qPCR was performed on StepOnePlus^TM^ platform (Thermo Fisher Scientific) using Fast SYBR^®^ Green Master Mix (Thermo Fisher Scientific). Gene expression in each sample was normalized to the expression of Gapdh. The sequences for primers used for RT-qPCR are as follows: Gapdh; 5′-CAT​GGC​CTT​CCG​TGT​TCC​TA-3′, 5′-GCC​TGC​TTC​ACC​ACC​TTC​TT-3′, Dot1l; 5′-TGG​CAA​GCC​TGT​CTC​CTA​CT-3′, 5′-CTG​CTC​CTC​CCT​GAG​TTT​TG-3′, Mmp3: 5′- ACA​TGG​AGA​CTT​TGT​CCC​TTT​TG-3′, 5′-TTG​GCT​GAG​TGG​TAG​AGT​CCC-3′ and Cldn1 5′-GGG​GAC​AAC​ATC​GTG​ACC​G-3′, 5′-AGG​AGT​CGA​AGA​CTT​TGC​ACT-3′.

### 2.4 RNA-seq analysis

For the RNA-sequencing (RNA-seq) analysis in the Cont and cKO BECs, pooled total RNAs isolated from BECs of 2–3 E15.5 pup skins, with RNA integrity number (RIN) greater than 7, were used to make a library for each genotype. The BEC RNAs were from the same embryos which the previously described LEC RNAs were extracted from (Yoo et al., 2020). For control and Dot1l overexpression BECs or LECs, cells from 2 to 3 T25 flask cultures were harvested to make libraries for each genotype. For RNA extraction, RNeasy Plus Mini Kit (Qiagen) was used according to the manufacturer’s instructions and total RNA isolated from BECs and LECs were used to prepare RNA-sequencing (RNA-seq) libraries using the ScriptSeq v2 kit (Illumina) according to the manufacturer’s instructions. Sequencing of the library was performed to produce single raw data for each genotype and cell type combination. RNA-seq data generated in the E15.5 Tie2-Cre(+); Dot1l^2f/2f^ and Tie2-Cre(−); Dot1l^2f/2f^ embryonic skin LECs (GSE104811) were downloaded and analyzed.

For post-sequencing data analysis, adapter sequences in the raw reads were trimmed with skewer (v0.2.2) (Jiang et al., 2014) with -L 75 and -e options specified. The skewer output reads were aligned to the mm10 UCSC mouse reference genome using a STAR read aligner (v2.7.9a) (Dobin et al., 2013). We quantified the mapped reads using VERSE (v0.1.5) (Zhu et al., 2016) with the -S option. Samples were normalized with the median of ratios method using the DESeq2 (v1.36.0) (Love et al., 2014) package in R (v4.2.0). Genes with normalized expression values ≥ 5 and values exceeding three times the value of the sample in comparison were defined as differentially expressed genes (DEGs) in Dot1l cKO samples, and those with normalized expression values ≥ 3 and values exceeding three times the value of the sample in comparison were defined as DEGs in OE samples. Genes with normalized expression values ≥ 5 and under three times the value of the sample in comparison were defined as unchanged genes (none-DEGs). DEG lists were used as inputs for GO enrichment analysis conducted on DAVID for GO terms in the biological pathway subset or KEGG pathways (v2022q2) (Huang da et al., 2009; Sherman et al., 2022). 10 GO terms or KEGG pathways with the lowest *p*-value were presented from each analysis. In Gene Set Enrichment Analysis (GSEA), “weighted” was selected for enrichment statistics and “log2 ratio of classes” was selected for metric of ranking genes (Subramanian et al., 2005). Gene expression change was presented using heatmap2 function of gplots R package (Warnes et al., 2022). Integrative genomic viewer (IGV) was used to verify the expression of the representative genes (Robinson et al., 2011).

### 2.5 ChIP-seq analysis

For chromatin immunoprecipitation-sequencing (ChIP-seq) analysis of H3K79me2 in LEC, the data was downloaded from Gene Expression Omnibus (GEO) under the accession code GSE104811. Adapters were trimmed from the raw reads using skewer (v0.2.2) (Jiang et al., 2014) using -L and -e options, and the reads were aligned to mm10 UCSC mouse genome using bowtie2 (v2.3.4.2) (Langmead and Salzberg, 2012). BedGraph files were constructed using MACS3 (v3.0.0b1) (Zhang et al., 2008) from the output of read alignment. The file was used to create H3K79me2 profile on the gene bodies using deepTools (v3.5.1) (Ramirez et al., 2016).

## 3 Results

### 3.1 Depletion of Dot1l transcripts in BECs leads to global transcriptomic changes

**FIGURE 1:**
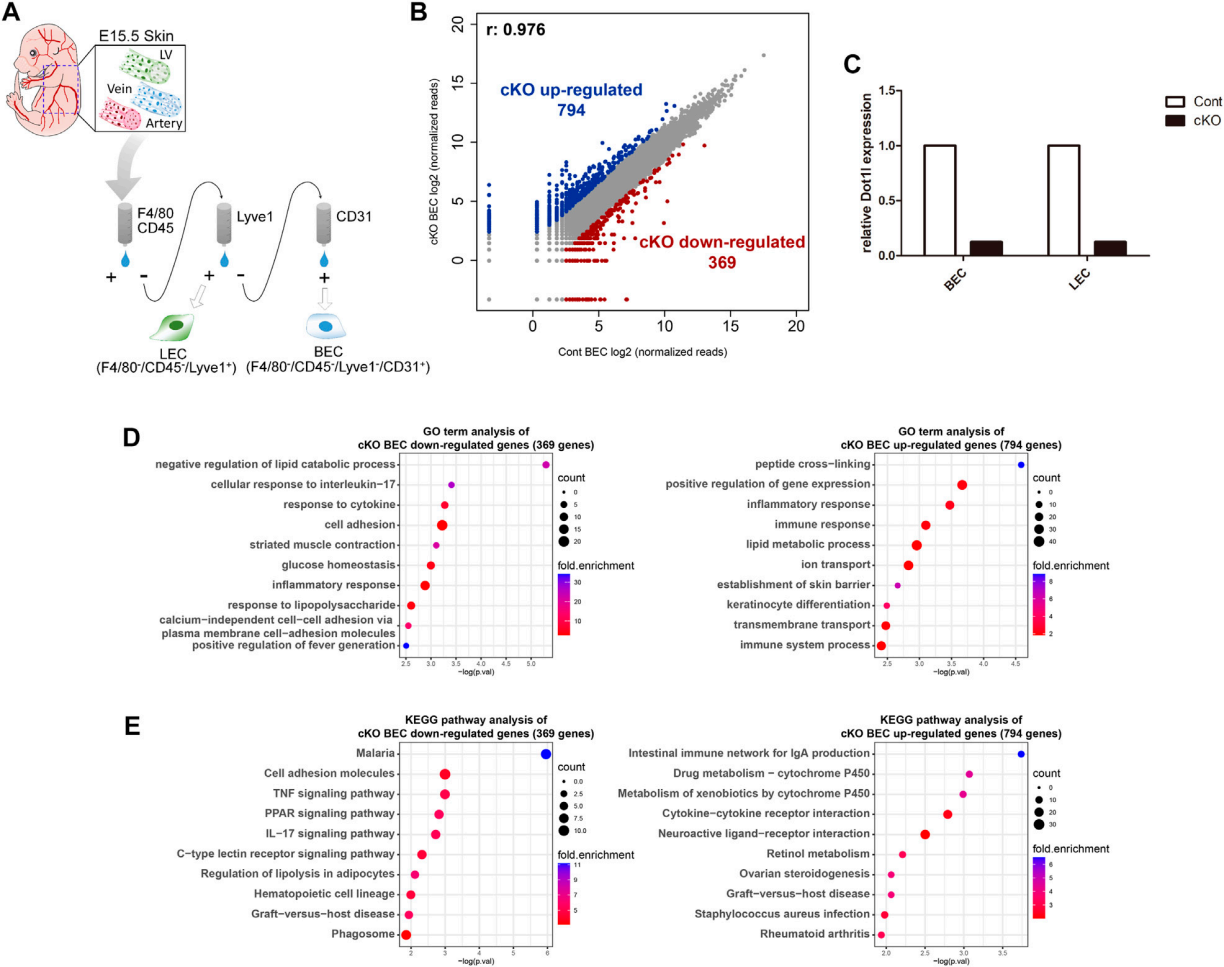
RNA-seq analysis of Dot1l cKO BECs. **(A)** Schematic diagram of the isolation of BECs from E15.5 cKO or control embryos. Cell surface markers used for BEC isolation were macrophage markers (F4/80/CD45)-negative, LEC marker (Lyve1)-negative, and BEC marker (CD31)-positive. **(B)** Scatterplot of differentially expressed genes (DEGs) according to a cutoff value, ≥5 normalized read counts, and fold change ≥3. **(C)** RT-qPCR results for validation of Tie2-Cre mediated Dot1l cKO. Expression of Dot1l is normalized to the expression value of Gapdh. (n = 1) **(D)** GO term analysis of down- and upregulated DEGs. **(E)** KEGG pathway analysis with down- and upregulated DEGs. Abbreviations: cKO, conditional knockout; LV, lymphatic vessel; LEC, lymphatic endothelial cell and BEC, blood endothelial cell.

To analyze Dot1l function in BECs, we performed RNA-sequencing (RNA-seq) analysis of Dot1l-depleted BECs isolated from E15.5 Tie2-Cre(+); Dot1l^2f/2f^ (conditional knockout, cKO) embryonic skin (Figure 1A). BECs isolated from E15.5 littermate Tie2-Cre(−); Dot1l^2f/2f^ embryonic skin were used as the control (Cont). As shown in Figure 1B, 794 genes were upregulated and 369 genes were downregulated in cKO BECs. Dot1l cKO was validated using RT-qPCR (Figure 1C). Gene ontology (GO) analysis of these DEGs in cKO BECs revealed associations between the downregulated genes and lipid catabolic process, cell adhesion, and immune response, and between the upregulation of genes and peptide cross-linking, positive regulation of gene expression, and immune response (Figure 1D). Pathway analysis of the DEGs showed that the genes involved in the tumor necrosis factor (TNF) signal-, peroxisome proliferator-activated receptor signal-, and interleukin (IL)17-related pathways were downregulated, and genes involved in drug metabolism and cytokine/receptor-related pathways were upregulated (Figure 1E).

### 3.2 Dot1l regulates common or distinct transcriptome in BECs and LECs

**FIGURE 2:**
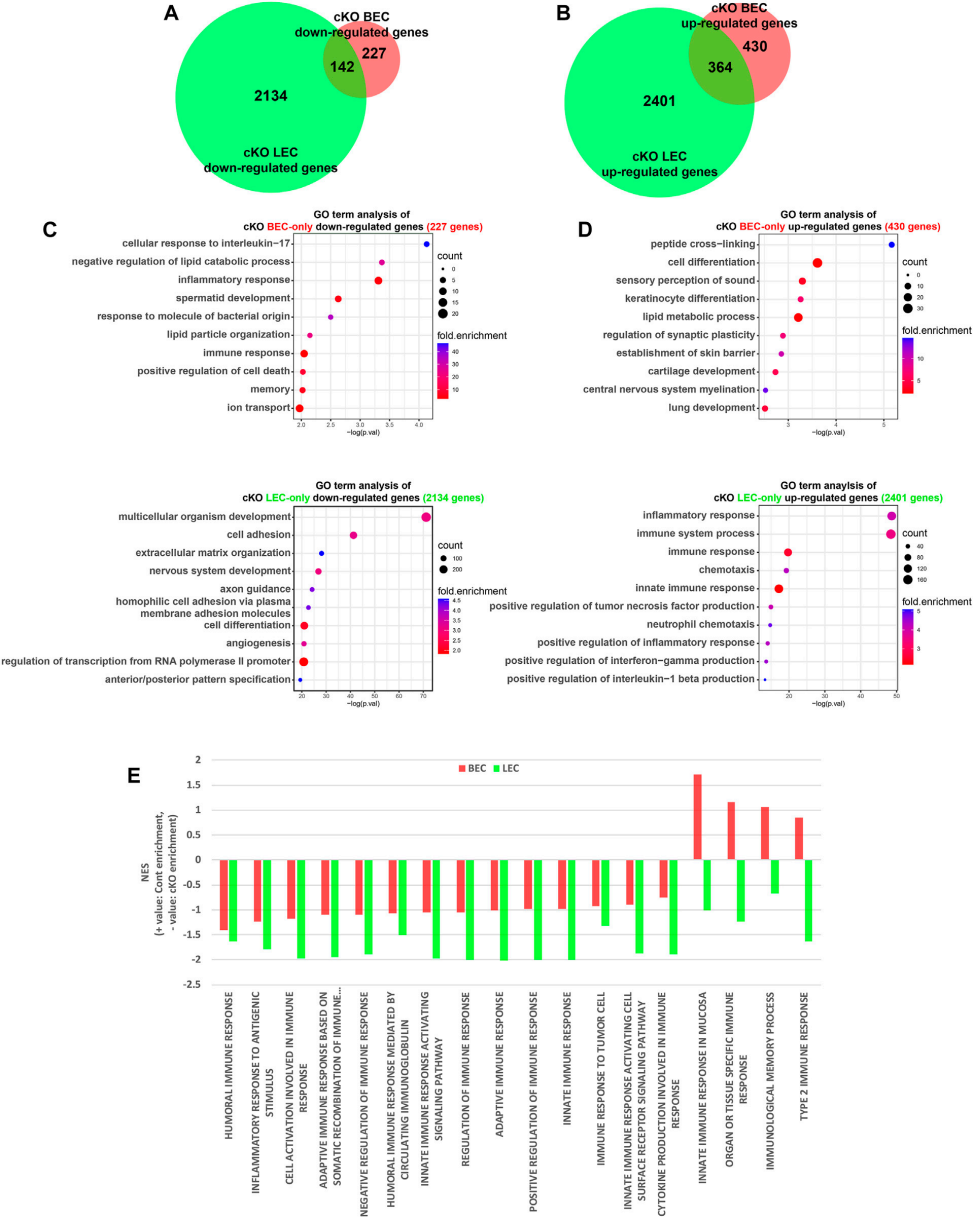
Comparison of DEGs between cKO BECs and LECs. **(A)** Venn diagram showing numbers of common and BEC- and LEC-only downregulated genes in cKO cells. **(B)** Venn diagram showing numbers of common and BEC- and LEC-only upregulated genes in the cKO. **(C)** GO terms identified for BEC- and LEC-only downregulated genes. **(D)** GO terms identified for BEC- and LEC-only upregulated genes. **(E)** Bar graph of normalized enrichment score (NES) of 18 child terms of the term “immune response”. Positive NES indicates enrichment in the control ECs and negative NES indicates enrichment in the Dot1l cKO ECs.

Next, we analyzed the regulatory function of Dot1l in the transcription of BECs and LECs. We previously showed that Dot1l loss represses genes involved in cell adhesion and angiogenesis in E15.5 skin LECs (Yoo et al., 2020). In this study, we compared the transcriptomic changes induced by Dot1l cKO in BECs and LECs (Figures 2A,B). The gene set repressed uniquely in the cKO LECs included genes related to cell adhesion, nervous system development, and angiogenesis (out of 2,134 genes) (Figure 2C). Meanwhile, genes related to immune response were enriched in cKO BECs repressed gene set (24 out of 227 genes, 10.6%; Figure 2C, Supplementary Figure S1A). The cKO LEC GO analysis of uniquely enhanced gene set also exhibited enrichment of immune response-related genes (375 out of 2,401 genes, 15.6%; Figure 2D). We further investigated expression pattern change of immunity-related genes using Gene Set Enrichment Analysis to identify which category of the immune system was up- or downregulated in each case (Subramanian et al., 2005). 18 child terms of the term “immune response” was obtained from the Amigo2 database (Carbon et al., 2009). Out of the 18 child terms, all 18 terms were significantly more upregulated in the Dot1l cKO LECs, while cKO BECs exhibited downregulation of “innate immune response in mucosa”, “organ or tissue specific immune response”, “immunological memory process” and “type 2 immune response” (Figure 2E; Supplementary Figure S2). This result attributes to decreased expression of genes such as *Il6*, *Arg2*, and *Irf1* in the cKO BECs (Supplementary Figure S1A).

To investigate whether the DEGs in Dot1l cKO ECs are a result of differential H3K79 methylation, LEC H3K79me2 ChIP data from our previous study was reanalyzed (Yoo et al., 2020). Reduction of H3K79me2 levels upon Dot1l inhibition by EPZ-5676 (also known as pinometostat) was observed in the gene bodies of commonly unchanged (none-DEGs) and downregulated DEGs but not in the commonly upregulated DEGs (Supplementary Figure S1C). The result provides additional evidence for association with the change of Dot1l-mediated H3K79 methylation and transcriptional activation. At the same time, the decrease of methylation level in the gene bodies of unchanged genes suggests that Dot1l is involved in transcription-independent cellular and molecular mechanisms.

The analysis of overlapping DEGs revealed 142 and 364 commonly repressed and elevated genes, respectively, in both ECs as a result of Dot1l depletion (Figures 2A,B). Analysis of GO terms revealed that downregulated genes were associated with glucose metabolism and upregulated genes were associated with cell-to-cell adhesion, and immunity-related terms (immune system process, inflammatory response, and others) (Supplementary Figure S1B). Representative genes visualized in the IGV tracks included *Cldn11* (downregulated in BECs and LECs), *CD84* (upregulated in BECs and LECs), *Socs3* (downregulated in BECs), *Spink5* (upregulated in BECs), *Cdh5* (downregulated in LECs), and *Tnf* (upregulated in LECs) (Supplementary Figure S1D).

### 3.3 Forced Overexpression of Dot1l transcripts changes distinct gene sets in BECs or LECs

**FIGURE 3:**
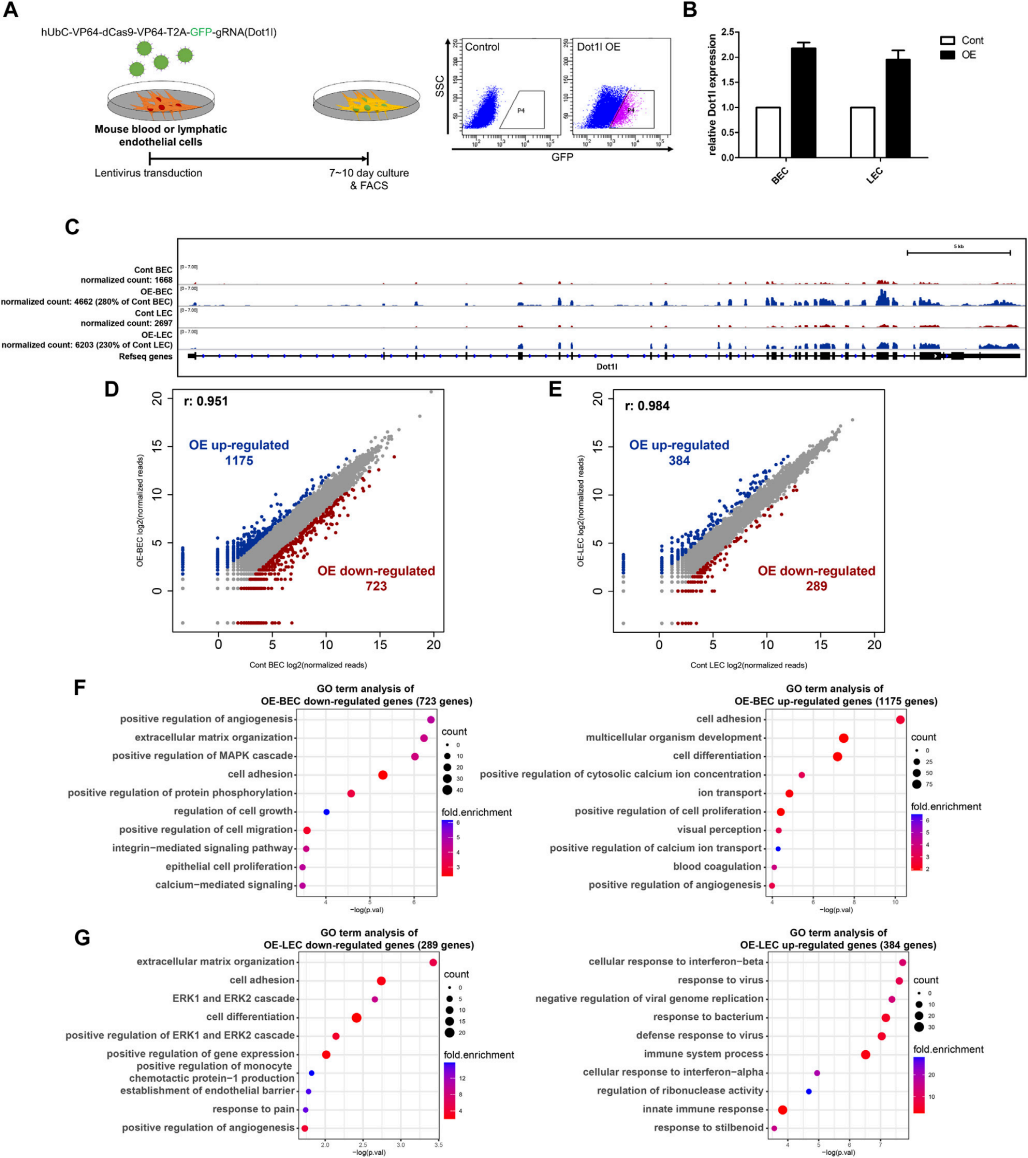
RNA-seq analysis in the Dot1l-overexpression (OE) BECs and LECs. **(A)** Schematic diagram showing lentivirus-mediated OE in either LECs or BECs via hUbC-VP64-dCas9-dCas9-T2A-GFP-gRNA (for Dot1l) vector and fluorescence-activated cell sorting of transduced cells (green fluorescent protein-positive [GFP+] cells). **(B)** RT-qPCR results for validation of lentiviral transduction mediated Dot1l OE. Dot1l expression is normalized to the expression value of Gapdh. (n = 2) **(C)** IGV track confirming Dot1l overexpression in LECs or BECs. **(D)** Scatter plot showing DEGs between control and OE-BECs. **(E)** Scatter plot showing DEGs between control and OE-LECs. Analysis of GO terms with down- or upregulated DEGs in OE-BECs **(F)** and OE-LECs **(G)**.

Next, we induced Dot1l overexpression in BECs (OE-BECs) or LECs (OE-LECs) to investigate its role in the regulation of gene transcription (Figure 3A). Overexpression of Dot1l and expression alteration of Mmp3 and Cldn1 in both types of cells were confirmed by RT-qPCR and in the IGV (Figures 3B,C; Supplementary Figures S3A, B). RNA-seq analysis identified 1,175 upregulated and 723 downregulated genes in OE-BECs, and 384 upregulated and 289 downregulated genes in OE-LECs (Figure 3D). Analysis of GO terms revealed OE-BECs had enhanced expression of genes associated with cell adhesion, development, and positive regulation of angiogenesis, and repressed expression of genes associated with positive regulation of angiogenesis, extracellular matrix (ECM) organization, cell adhesion, and cell proliferation. OE-LECs displayed enhanced transcriptional activity of genes associated with cellular response to interferon-beta and response to virus and immune system processes, and repressed activity of genes associated with ECM organization, cell adhesion, extracellular signal-regulated kinase (ERK)1 and ERK2 cascade, and establishment of the endothelial barrier (Figures 3F,G).

Next, we sought to identify genes that are highly dependent on Dot1l for their expression by comparing gene expression change between the cKO and OE samples. To that end, genes with normalized expression values that were either more than three times higher or less than one-third of the expression value in the control were selected. In total, there were 16 genes for BECs and 22 genes for LECs (Supplementary Figure S3C). Notably, GO term analysis in the cKO vs. OE DEGs of LECs revealed an enrichment of genes related to cell adhesion and blood vessel remodeling among the selected genes (Supplementary Figure S3D). The genes Stab2, Fat2, Emp2, and Sulf1 were associated with the term cell adhesion, and Sema3c and Axl were associated with the term blood vessel remodeling.

### 3.4 Comparison of transcriptomic alterations between OE-BECs and OE-LECs

**FIGURE 4:**
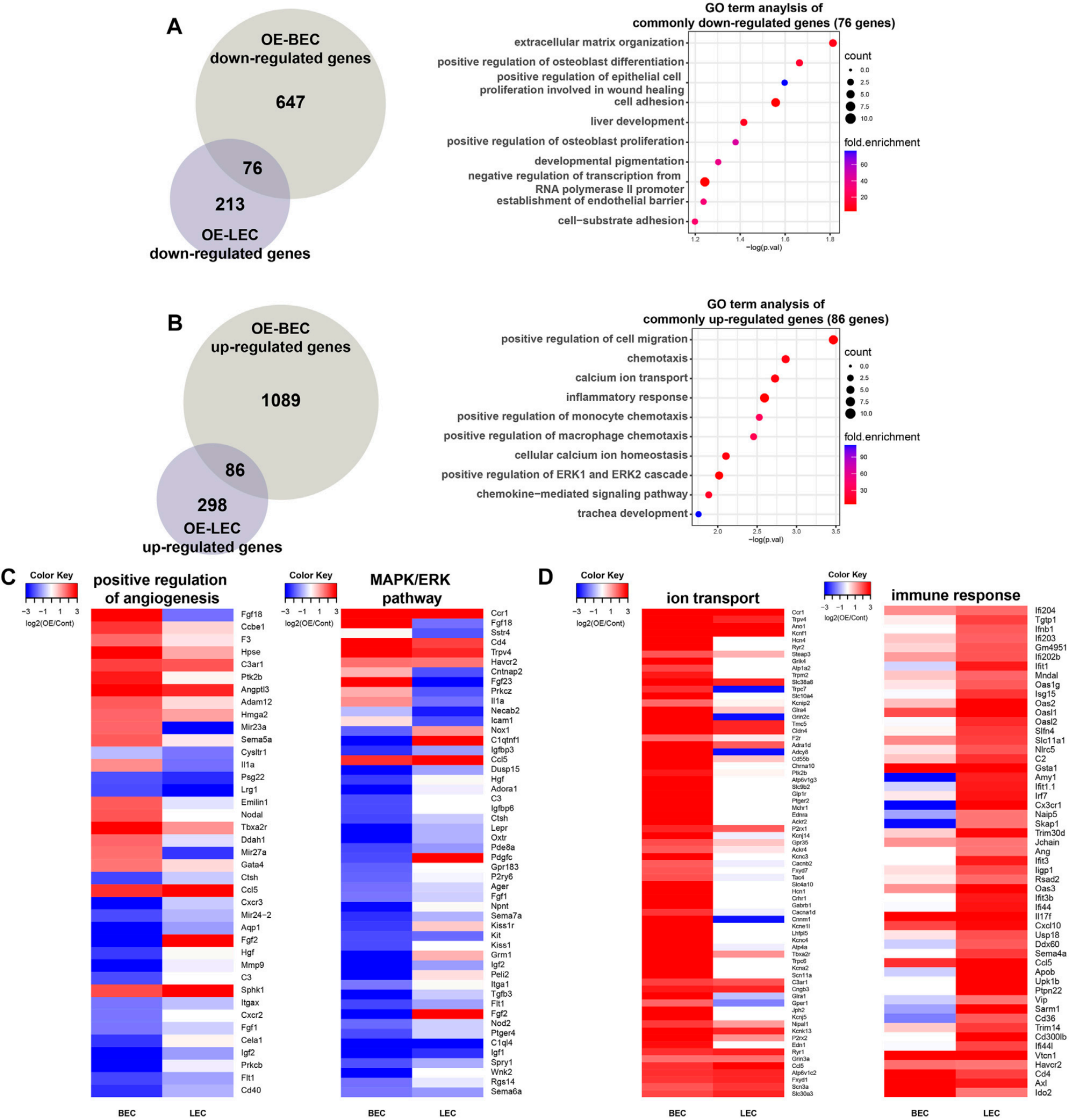
Comparison of DEGs between OE-BECs and OE-LECs. **(A)** Venn diagram showing numbers of common and BEC- and LEC-only downregulated genes in an analysis of GO terms. **(B)** Venn diagram showing numbers of common and BEC- and LEC-only upregulated genes in an analysis of GO terms. **(C)** Heatmap representing expression change of genes related to positive regulation of angiogenesis and MAPK/ERK pathway. **(D)** Heatmap representing expression change of genes related to ion transport and immune response.

We then compared genes that were up- or downregulated in OE-BECs and OE-LECs (Figures 4A,B). As shown in Figure 4A, out of a total of 76 genes, genes associated with ECM organization, differentiation, EC proliferation involved in wound healing, and cell adhesion were commonly downregulated in OE-BECs and OE-LECs (Figure 4A). Of a total of 86 genes, those associated with positive regulation of cell migration, chemotaxis, and inflammatory response were commonly upregulated in OE-BECs and OE-LECs (Figure 4B). Interestingly, common and related terms, such as positive regulation of angiogenesis, extracellular matrix organization, cell adhesion, positive regulation of MAPK cascade, and ERK1 and ERK2 cascade, were found in the most significantly enriched terms in downregulated DEG sets of both types of OE-ECs (BEC: 38 out of 723 genes, 5.3%; LEC: 28 out of 289 genes, 9.7%; Figures 3E,F). The term positive regulation of ERK1 and ERK2 cascade were also enriched in GO term analysis results of the commonly upregulated gene set of both Dot1l OE-ECs (5 out of 86 genes, 5.8%; Figures 4B,C). This implies a close connection between Dot1l-mediated transcriptional regulation and MAPK/ERK signaling pathway during EC development. In the analysis result of upregulated genes, OE-BEC upregulated genes are enriched in GO terms such as cell adhesion and ion transport, while OE-LEC upregulated genes are enriched in immunity related GO terms (Figures 3F,G). GO term analysis of the overlapping DEGs from each comparison presented enrichment in both calcium ion transport and inflammatory response terms (Figures 4B,D).

## 4 Discussion

Dot1l has critical roles in cardiovascular development and function. Dot1l loss leads to defects in heart development and function *in vivo* (Nguyen et al., 2011), LEC development and function *in vivo* (Yoo et al., 2020), and BEC function *in vitro* (Duan et al., 2016). Using genetic and epigenomic approaches, we previously provided evidence that Dot1l is essential for LEC differentiation from the hemogenic endothelium and takes part in the transcriptional regulation process. Consistent with our previous study, another study showed that Dot1l is a critical factor that regulates human LEC migration (Williams et al., 2017). A genome-wide study showed that LECs require multiple-layered signaling pathways systemically regulated by each other. However, the function of Dot1l in BECs, especially function in transcriptional regulation, remains unclear. Furthermore, to the best of our knowledge, no study has directly compared the transcriptomes of BECs and LECs in which epigenetic factors are depleted. However, it has been clearly shown that epigenetic mechanisms involved in establishing, diversifying, and maintaining endothelial function depend on their location in different organs, their function, and cell type (BEC vs*.* LEC) (Bronneke et al., 2012; Yan and Marsden, 2015; Schlereth et al., 2018; Nakato et al., 2019; Gutierrez et al., 2021; Tacconi et al., 2021).

A well-known regulatory function of Dot1l in gene transcription is mediated through H3K79 methylation. The H3K79 methylation promotes the euchromatin state and consequent transcription factor recruitment and transcription initiation (Steger et al., 2008; Wang et al., 2008; Sarno et al., 2020). Using the previous H3K79me2 ChIP-seq analysis in cKO LECs after EPZ-5676 treatment, we found decrease in methylation level of gene bodies of Dot1l cKO common downregulated DEGs (Supplementary Figure S1C). H3K79 di-/tri-methylation is strongly correlated with active gene transcription (Steger et al., 2008) and other active histone markers in mammalian cells. Dot1l knockdown impaired the differentiation potential of KIND1 and HES3 cells by reducing NKX2.5 expression (Pursani et al., 2018) and Dot1l cKO caused lymph vessel development failure by reducing Sox18, Vegfr3, Ramp2, Foxc2, Efnb2, and Eph4 expression. On the other hand, Dot1l-mediated transcriptional repression has also been reported in a number of studies. H3K79me3 has been demonstrated to localize more at silent promoters than active promoters except for narrow region surrounding TSS (Barski et al., 2007). Dot1l-depleted hematopoietic progenitor cells (HPCs) exhibited increased expression of genes involved in HPC differentiation induction (Borosha et al., 2022). Non-cardiomyocyte related genes were increased in Dot1l KO neonatal cardiomyocytes (Cattaneo et al., 2022). GO term analysis of SGC0946 Dot1l inhibitor treatment or Dot1l KO upregulated genes found enrichment of inflammatory response and related terms in macrophages (Willemsen et al., 2022). Consistently, our analysis suggests repressive activity of Dot1l by demonstrating that Dot1l cKO can induce increased gene expression and Dot1l OE can induce reduced gene expression. Specifically, genes associated with immune responses were increased in Dot1l cKO BECs and LECs and genes associated with cell adhesion was repressed in OE-BECs and OE-LECs (Supplementary Figure S1B; Figure 4A). Furthermore, the decrease of H3K79 methylation level in the gene bodies of common none-DEGs suggests that Dot1l could be involved in transcription independent functions such as DNA repair (Huyen et al., 2004) and increased H3K79me2 level does not necessarily result in increased gene transcription (Supplementary Figure S1C). Further research is required to identify factors working in conjunction with Dot1l or Dot1l-mediated H3K79 methylation in transcription regulation.

Neighboring ECs are tightly held together via the expression of various types of adhesion molecules by the cells. These include tight junction species, including junctional adhesion molecules, EC-selective adhesion molecules, and claudins, as well as adherens junction species that include vascular endothelial cadherin (Dejana et al., 2009; Giannotta et al., 2013; Reglero-Real et al., 2016). Furthermore, cell adhesion molecules, such as claudin 1 (CLDN1) and CLDN11, are differentially expressed in an organotypic manner (Nakato et al., 2019). It is interesting to note that CLDN11 expression was repressed in both cKO BECs and cKO LECs. These DEGs are most likely mediated by differential epigenetic marks and/or priming (Matouk and Marsden, 2008; Turgeon et al., 2020). Enrichment of GO term hemophilic cell adhesion via plasma membrane adhesion molecules was also highlighted in our analysis of genes diminished in both cKO EC types. Protocadherin beta 2 (Pcdhb2), Pcdhb18, Pcdhb, Pcdhb3, Pcdhb12, Pcdhb11, and Pcdhb10 genes were found to be involved (Supplementary Figure S1A). The PCDH family is a member of the cadherin superfamily and is essential for neural development (Sano et al., 1993; Hayashi and Takeichi, 2015). PCDHs can be divided into two subgroups: clustered and non-clustered PCDHs (Colas-Algora and Millan, 2019). The genes encoding clustered PCDHs include the PCDHα, β, and γ families and they are located closely to each other within a small region in the genome, while the genes encoding non-clustered PCDHs are dispersed throughout the genome. In our analysis, the expression change was prominent in the PCDH β family. The function of PCDHB genes should be further explored, as no study has clearly provided their function in EC development.

Owing to their location in vessels, ECs are continuously exposed to pathogens, immunogenic signals, or stimuli (Amersfoort et al., 2022). Therefore, it is important that ECs promptly respond to these signals via gene transcription. Evidence suggests that a subset of ECs acts as immune cells by modulating immunity (Pober and Sessa, 2007; Shao et al., 2020). Furthermore, some ECs display typical features of immune cells, in which ECs express co-inhibitory and co-stimulatory receptors (Carman and Martinelli, 2015), induce apoptosis of tumor cells (Motz et al., 2014), secrete cytokines, and act as antigen-presenting cells (Daar et al., 1984a; Daar et al., 1984b). Endothelial hepatitis A virus cellular receptor 2 that is upregulated in both cKO ECs enhances defense mechanism against *Rickettsia heilongjiangensis* (Yang et al., 2016).

In our analyses, cell migration was ranked as the most enriched GO term in the analysis of commonly upregulated genes in OE BECs and LECs. C-X-C motif chemokine ligand 10, one of the upregulated genes in OE-Dot1l BECs and LECs, promotes the migration of cardiac microvascular ECs (CMEC), likely by regulating the p38/focal adhesion kinase pathways without changing CMEC proliferation and viability (Xia et al., 2016). A recent study showed that LETR1, an LEC-dominant long noncoding RNA, modulates the expression of semaphorin 3C, one of the upregulated genes in OE-Dot1l BECs and LECs, and promotes the growth and migration of LECs by changing chromatin structure (Ducoli et al., 2021). Williams et al. have performed a series of siRNA screening assays by targeting individual protein-coding genes and identified 111 genes critical for BEC and/or LEC migration (Williams et al., 2017). We have found that the mouse homologs of their screening results were present in our DEG list. Among the overlapping genes, Cwc22 has been identified to be downregulated in both cKO ECs but not in the OE ECs, and Hpdl and Ugt1a9 were upregulated in both OE ECs and not in the cKO ECs. On the other hand, Abcc3 and Gcgr were upregulated in both cKO ECs and not in the OE ECs. Cwc22, Hpdl and Ugt1a9 represent the transcriptional activation, and Abcc3 and Gcgr represents the transcriptional repression mediated by Dot1l in genes related to cell migration (data not shown). Furthermore, using the mouse LEC H3K79me2 ChIP data from our previous research, we have confirmed that H3K79me2 levels in their gene bodies reduce when Dot1l function is inhibited by EPZ-5676, providing evidence for Dot1l regulated transcription (data not shown). Genes which do not show a Dot1l-dependent expression pattern are expected to be regulated by Dot1l-independent mechanism or represent the difference between the mouse and human ECs.

Positive regulation of the ERK1/ERK2 cascade was identified as the one of the most significantly enriched GO term among the uniquely downregulated genes of OE-LECs (Figure 3F). VEGFR3 signaling activates ERK1/2 signaling and induces LEC proliferation (Qin et al., 2021). Interestingly, OE-Dot1l in BECs selectively led to repression of genes related to positive regulation of angiogenesis, whereas OE-Dot1l in the LECs induced a set of genes involved in the function of cell adhesion. In contrast, cellular response to interferon-beta related genes were enriched in OE-LECs upregulated gene set. The comprehensive analysis demonstrates EC type-dependent transcriptional regulation of angiogenesis-related genes by Dot1l. Histone modifications are significant contributors to the regulation of angiogenic gene expression. A recent study clearly showed common (or core) and diverse gene signatures and epigenetic landscapes across developmental time and organotypic space in developing mouse and adult BECs (Gutierrez et al., 2021). Our study further demonstrates that a single epigenetic factor can differentially function and build-up unique chromatin structure to regulate gene transcription even in the developmentally close cells. Recently, Kanki et al. (2022) showed that the genes of immediate-early angiogenic transcription factors exclusively acquire protein regulator of cytokinesis (PRC)1.3/PRC2-mediated bivalent histone markers (both H3K4me3-and H3K27me3-positive) after VEGF stimulus. In addition, Ezh2, a component of the PRC2 complex, inhibits the endothelial expression of Fosl1, Creb3l1, Klf5, and Mmp9 to maintain vascular integrity during embryonic development (Delgado-Olguin et al., 2014). Lysine demethylase 3A (Kdm3a) expression is induced in BECs by hypoxia. Its elevated expression accelerates matrix metalloproteinase 12 expression via histone H3K9 demethylation and facilitates trophoblast invasion and uterine vascular remodeling (Chakraborty et al., 2016). The Jmjd6 histone arginine demethylase regulates the expression and splicing of the angiogenic factor Flt1 and controls the angiogenic sprouting of BECs (Boeckel et al., 2011).

Gene OE experiments frequently result in suboptimal physiological protein levels, potentially enabling the formation of transcriptional complexes that would not typically occur (Moriya, 2015). Nevertheless, ECs in our OE experiments exhibited 2∼3-fold upregulation of Dot1l expression, suggesting that it falls within the physiological range (Figure 3B). Therefore, the result of our study can be used for understanding the Dot1l-mediated transcription regulation. As for Dot1l expression in disease condition, it was shown to potentially contribute to the development of multiple cancers including leukemia (Okada et al., 2005; Bernt et al., 2011), breast cancer (Cho et al., 2015; Nassa et al., 2019), ovarian cancer (Zhang et al., 2017), prostate cancer (Vatapalli et al., 2020), and other cancers (Jacinto et al., 2009; Campbell et al., 2016; Zhu et al., 2018; Liu et al., 2020). Given that tumor metastasis is positively or negatively mediated by lymphatics (Dieterich et al., 2022; La et al., 2022), the identified genes regulated by Dot1l can be targets for the detection or prevention of cancer progression. Indeed, studies suggest that Dot1l itself can serve as a prognostic biomarker for ovarian cancer and gastric cancer (Zhang et al., 2017; Song et al., 2020), and pharmacological inhibition of its activities with EPZ-5676 has a beneficial effect in patients with mixed lineage leukemia (Stein et al., 2018).

In the present study, we directly compared the capability of mouse Dot1l in the regulation of gene transcription in BECs and LECs. Common and distinct sets of genes regulated by Dot1l were identified depending on EC types and H3K79me2 levels in LEC has also provided insight into the relationship between epigenetic modification and transcriptional regulation. To the best of our knowledge, this is the first study to delineate the regulatory functions of a specific epigenetic factor in two different ECs, in which its function is abolished or strengthened. Nevertheless, analysis of H3K79 methylation in BECs and investigation of histone methylation-independent transcriptional change in aberrant Dot1l expression could provide more thorough understanding of molecular mechanism governing EC type-specific functions. Additionally, follow-up studies on changes and differences in transcriptomic and epigenetic landscapes in developing and adult ECs in multiple organs would be helpful in precisely understanding the cellular heterogeneity of ECs.

## Data Availability

The original contributions presented in the study are publicly available. This data can be found here: [GSE214945]. Publicly available datasets were analyzed in this study. This data can be found here: [GSE104811]. Links to the repositories are as follows GSE214945: https://www.ncbi.nlm.nih.gov/geo/query/acc.cgi?acc=GSE214945, GSE104811: https://www.ncbi.nlm.nih.gov/geo/query/acc.cgi?acc=GSE104811.

## Supplementary material

SUPPLEMENTARY FIGURE S1
**(A)** Heatmap representing expression change of genes under the GO term immune response, glucose metabolism and cell-to-cell adhesion. **(B)** GO terms identified for commonly up- or down-regulated genes. **(C)** H3K79me2 profiles on DMSO and EPZ-5676 treated LEC. The gene body profiles of none-DEGs, down- and up-regulated DEGs are shown. **(D)** IGV visualization of Cont and cKO EC RNA-seq for representative genes.

SUPPLEMENTARY FIGURE S2 Results of Cont and cKO EC comparison in 18 child terms of the term “immune response” shown in GSEA enrichment plot.

SUPPLEMENTARY FIGURE S3
**(A)** RT-qPCR results of Mmp3 and Cldn1. Gene expression is normalized to the expression value of Gapdh in Cont and OE EC. (n=2) **(B)** IGV track visualization of Cont and OE EC RNA-seq for Mmp3 and Cldn1. **(C)** Heatmap representing expression change of Dot1l dependent genes. **(D)** Upper panel shows the GO term analysis results for the Dot1l dependent genes in the BECs and the lower panel shows the GO term analysis results for the LECs.
