# Supplementary figures and images for: Common and distinct functions of mouse Dot1l in the regulation of endothelial transcriptome
Source: Front Cell Dev Biol. 2023 Jun 15;11:1176115. doi: 10.3389/fcell.2023.1176115 (PMC10311421; doi:10.3389/fcell.2023.1176115)

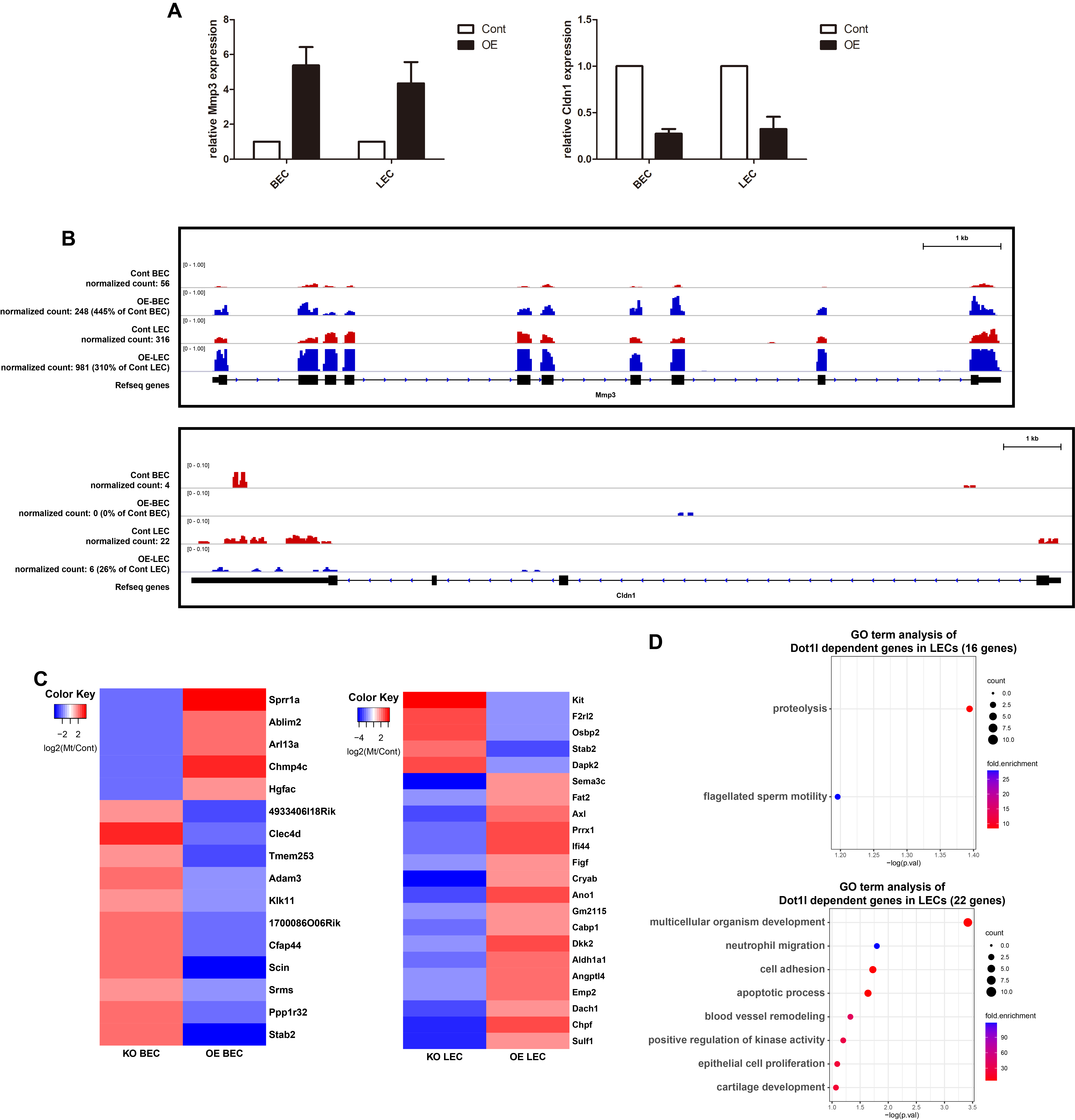

Supplement: Supplementary file 1 [file Image3.JPEG]

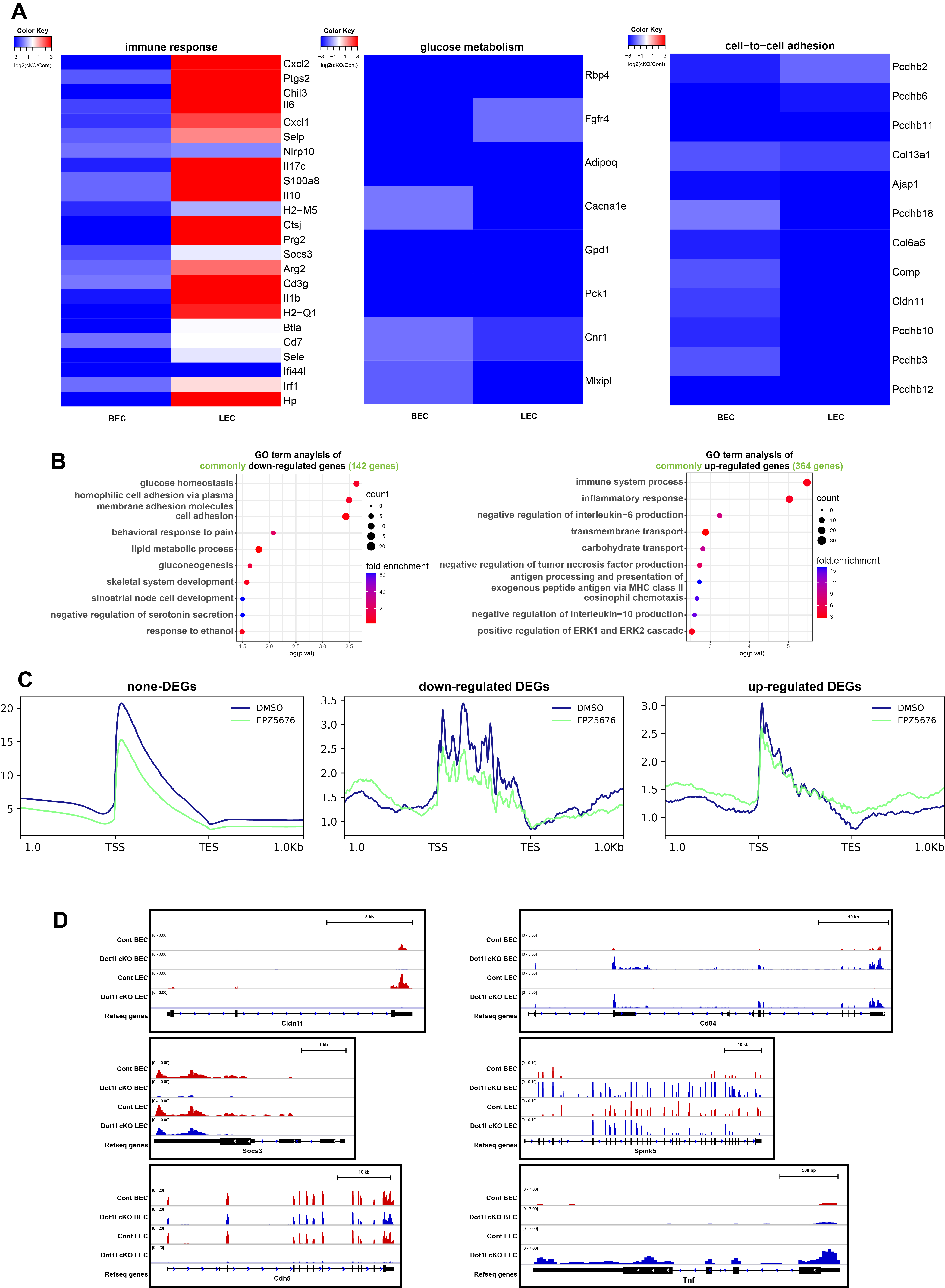

Supplement: Supplementary file 2 [file Image1.JPEG]

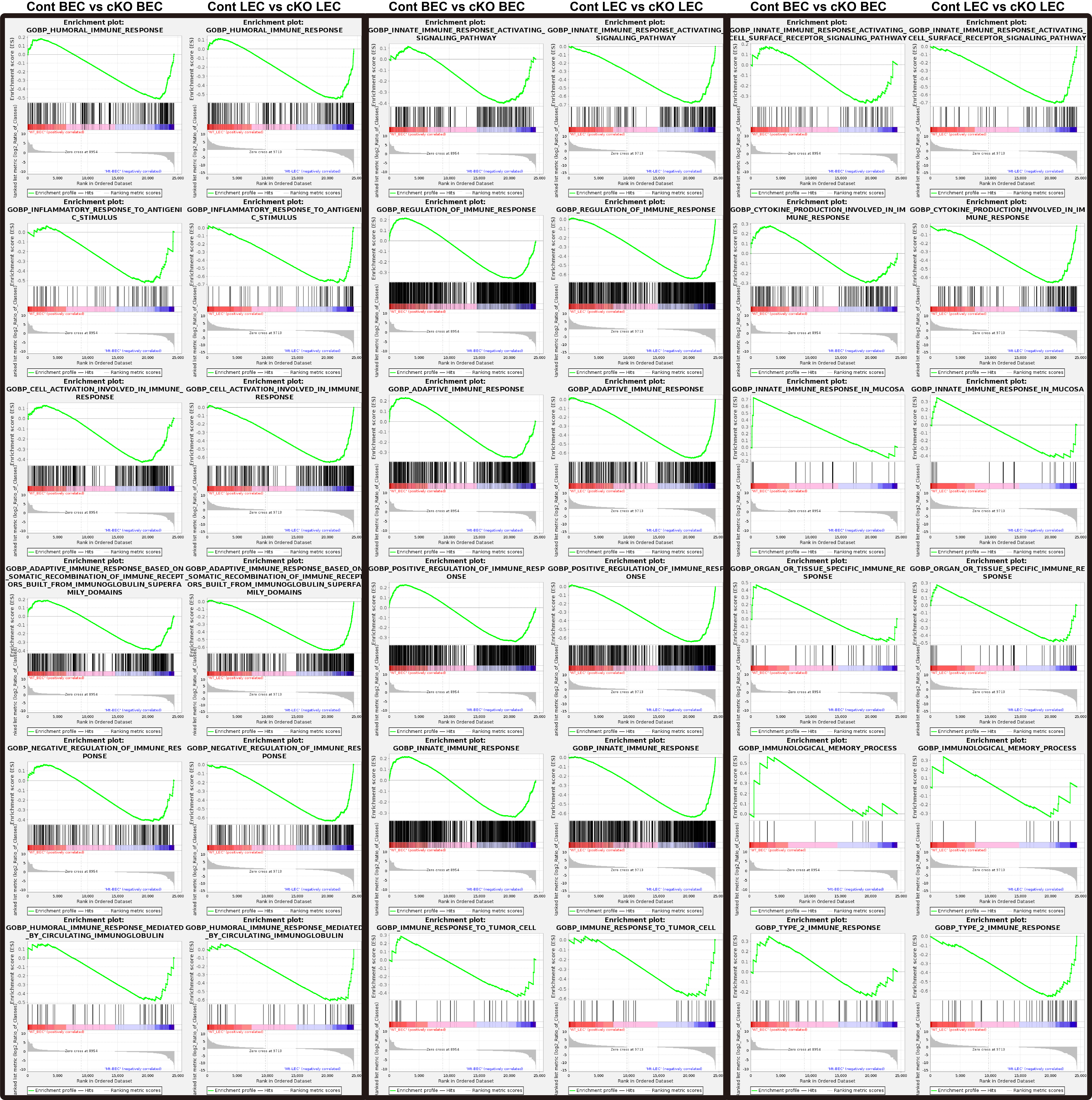

Supplement: Supplementary file 3 [file Image2.JPEG]
